# *e*ToxPred: a machine learning-based approach to estimate the toxicity of drug candidates

**DOI:** 10.1186/s40360-018-0282-6

**Published:** 2019-01-08

**Authors:** Limeng Pu, Misagh Naderi, Tairan Liu, Hsiao-Chun Wu, Supratik Mukhopadhyay, Michal Brylinski

**Affiliations:** 1 Division of Electrical & Computer Engineering, Louisiana State University, Baton Rouge, LA 70803 USA; 2 Department of Biological Sciences, Louisiana State University, Baton Rouge, LA 70803 USA; 3 Department of Mechanical Engineering, Louisiana State University, Baton Rouge, LA 70803 USA; 4 Department of Computer Science, Louisiana State University, Baton Rouge, LA 70803 USA; 5 Center for Computation & Technology, Louisiana State University, Baton Rouge, LA 70803 USA

**Keywords:** Virtual screening, Synthetic accessibility, Toxicity, Machine learning, Deep belief network, Extremely randomized trees

## Abstract

**Background:**

The efficiency of drug development defined as a number of successfully launched new pharmaceuticals normalized by financial investments has significantly declined. Nonetheless, recent advances in high-throughput experimental techniques and computational modeling promise reductions in the costs and development times required to bring new drugs to market. The prediction of toxicity of drug candidates is one of the important components of modern drug discovery.

**Results:**

In this work, we describe *e*ToxPred, a new approach to reliably estimate the toxicity and synthetic accessibility of small organic compounds. *e*ToxPred employs machine learning algorithms trained on molecular fingerprints to evaluate drug candidates. The performance is assessed against multiple datasets containing known drugs, potentially hazardous chemicals, natural products, and synthetic bioactive compounds. Encouragingly, *e*ToxPred predicts the synthetic accessibility with the mean square error of only 4% and the toxicity with the accuracy of as high as 72%.

**Conclusions:**

*e*ToxPred can be incorporated into protocols to construct custom libraries for virtual screening in order to filter out those drug candidates that are potentially toxic or would be difficult to synthesize. It is freely available as a stand-alone software at https://github.com/pulimeng/etoxpred.

## Background

Drug discovery is an immensely expensive and time-consuming process posing a number of formidable challenges. To develop a new drug requires 6–12 years and costs as much as $2.6 billion [1, 2]. These expenses do not include the costs of basic research at the universities focused on the identification of molecular targets, and the development of research methods and technologies. Despite this cumbersome discovery process, the pharmaceutical industry is still regarded as highly profitable because the expenses are eventually accounted for in the market price of new therapeutics. Although, a breakdown of the overall capitalized costs shows that the clinical period costing $1.5 billion is economically the most critical factor, the expenditures of the pre-human phase aggregate to $1.1 billion [1]. Thus, technological advances in discovery research and preclinical development could potentially lower the costs of bringing a new drug to the market.

Computer-aided drug discovery (CADD) holds a significant promise to reduce the costs and speed up the development of lead candidates at the outset of drug discovery [3]. Powered by continuous advances in computer technologies, CADD employing virtual screening (VS) allows identifying hit compounds from large databases of drug-like molecules much faster than traditional approaches. CADD strategies include ligand- and structure-based drug design, lead optimization, and the comprehensive evaluation of absorption, distribution, metabolism, excretion, and toxicity (ADMET) parameters [4]. Ligand-based drug design (LBDD) leverages the spatial information and physicochemical features extracted from known bioactives against a given target protein to design and optimize new compounds for the same target [5]. VS employing features provided by pharmacophore modeling [6] and quantitative structure-activity relationship (QSAR) analysis [7] can be performed in order to identify potentially active compounds. Although the capabilities of the traditional LBDD to discover new classes of leads may be limited, recent advances in generating targeted virtual chemical libraries by combinatorial chemistry methods considerably extend the application of LBDD methods [8–10]. Captopril, an angiotensin-converting enzyme inhibitor, was one of the first success stories of LBDD, which was considered a revolutionary concept in 1970s compared to conventional methods [11].

Although the combination of pharmacophore modeling, QSAR, and VS techniques has been demonstrated to be valuable in the absence of the protein structure data [12, 13], the three-dimensional (3D) information on the target protein allows employing structure-based drug design (SBDD) [14] in CADD. Foremost SBDD methods include molecular docking [15], molecular dynamics [16], receptor-based VS [17], and the de novo design of active compounds [18]. Molecular docking is widely used in CADD to predict the preferable orientation of a drug molecule in the target binding pocket by finding the lowest energy configuration of the protein-ligand system. It is often employed to conduct receptor-based VS whose goal is to identify in a large library of candidate molecules those compounds that best fit the target binding site. VS performed with high-performance computing machines renders docking programs such as AutoDock Vina [19], rDock [20], Glide [21], and FlexX [22] capable to search through millions of compounds in a matter of days or even hours. A potent, pyrazole-based inhibitor of the transforming growth factor-β type I receptor kinase exemplifies benefits of utilizing receptor-based VS to discover leads. This inhibitor has been independently discovered with the computational, shape-based screening of 200,000 compounds [23] as well as the traditional enzyme and cell-based high-throughput screening of a large library of molecules [24].

In addition to LBDD and SBDD, toxicity prediction is an increasingly important component of modern CADD, especially considering that the collections of virtual molecules for VS may comprise tens of millions of untested compounds. Methods to predict toxicity aim at identifying undesirable or adverse effects of certain chemicals on humans, animals, plants, or the environment. Conventional approaches to evaluate toxicity profiles employing animal tests are constrained by time, costs, and ethical considerations. On that account, fast and inexpensive computational approaches are often employed at first in order to eliminate potentially toxic molecules and reduce the number of experimental tests that need to be conducted. For instance, a blockage of the human Ether-à-go-go-Related Gene (hERG) potassium ion channels by a surprisingly diverse group of drugs can induce lethal cardiac arrhythmia [25]. Therefore, the effective identification of putative hERG blockers and non-blockers in chemical libraries plays an important role in the cardiotoxicity prediction. A recently developed method, Pred-hERG, estimates the cardiac toxicity with a set of features based on statistically significant and externally predictive QSAR models of the hERG blockage [26]. Pred-hERG employs a binary model, a multi-class model, and the probability maps of atomic contribution, which are combined for the final prediction. Encouragingly, Pred-hERG achieves a high correct classification rate of 0.8 and a multi-class accuracy of 0.7.

Another example is chemTox (http://www.cyprotex.com/insilico/physiological_modelling/chemtox) predicting key toxicity parameters, the Ames mutagenicity [27] and the median lethal dose (LD_50_) following intravenous and oral administration, as well as the aqueous solubility. chemTox employs molecular descriptors generated directly from chemical structures to construct quantitative-structure property relationships (QSPR) models. Since this method requires a set of specific descriptors to generate QSPR models for a particular type of toxicity, it may not be suitable to evaluate a broadly defined toxicity and drug side-effects in general. A similar method, ProTox, predicts rodent oral toxicity based on the analysis of toxic fragments present in compounds with known LD_50_ values [28]. ProTox additionally evaluates possible targets associated with adverse drug reactions and the underlying toxicity mechanisms with the collection of protein-ligand pharmacophores, called toxicophores. This tool was reported to outperform the commercial software TOPKAT (TOxicity Prediction by Komputer Assisted Technology, http://accelrys.com/products/collaborative-science/biovia-discovery-studio/qsar-admet-and-predictive-toxicology.html) against a diverse external validation set, with the sensitivity, specificity and precision of 0.76, 0.95 and 0.75, respectively. Other techniques to predict toxicity utilize various features such as fingerprints, physicochemical properties, and pharmacophore models to build predictive dose- and time-response models [29].

The Tox21 Data Challenge 2014 (https://tripod.nih.gov/tox21/challenge/index.jsp) has been conducted to assess a number of methods predicting how chemical compounds disrupt biological pathways in ways that may result in toxic effects. In this challenge, the chemical structure data for 12,707 compounds were provided in order to evaluate the capabilities of modern computational approaches to identify those environmental chemicals and drugs that are of the greatest potential concern to human health. DeepTox [30] was the best performing methods in the Tox21 Data Challenge winning the grand challenge, the nuclear receptor panel, the stress response panel, and six single assays. This algorithm employs the normalized chemical representations of compounds to compute a large number of descriptors as an input to machine learning. Models in DeepTox are first trained and evaluated, and then the most accurate models are combined into ensembles ultimately used to predict the toxicity of new compounds. DeepTox was reported to outperform deep neural networks (DNNs) [31], support vector machines (SVMs) [32], random forests (RF) [33], and elastic nets [34].

In this communication, we describe *e*ToxPred, a new method to predict the synthetic accessibility and the toxicity of molecules in a more general manner. In contrast to other approaches employing manually-crafted descriptors, *e*ToxPred implements a generic model to estimate the toxicity directly from the molecular fingerprints of chemical compounds. Consequently, it may be more effective against highly diverse and heterogeneous datasets. Machine learning models in *e*ToxPred are trained and cross-validated against a number of datasets comprising known drugs, potentially hazardous chemicals, natural products, and synthetic bioactive compounds. We also conduct a comprehensive analysis of the chemical composition of toxic and non-toxic substances. Overall, *e*ToxPred quite effectively estimates the synthetic accessibility and the toxicity of small organic compounds directly from their molecular fingerprints. As the primary application, this technique can be incorporated into high-throughput pipelines constructing custom libraries for virtual screening, such as that based on *e*MolFrag [9] and *e*Synth [10], to eliminate from CADD those drug candidates that are potentially toxic or would be difficult to synthesize.

## Implementation

### Machine learning algorithms

**Fig. 1:**
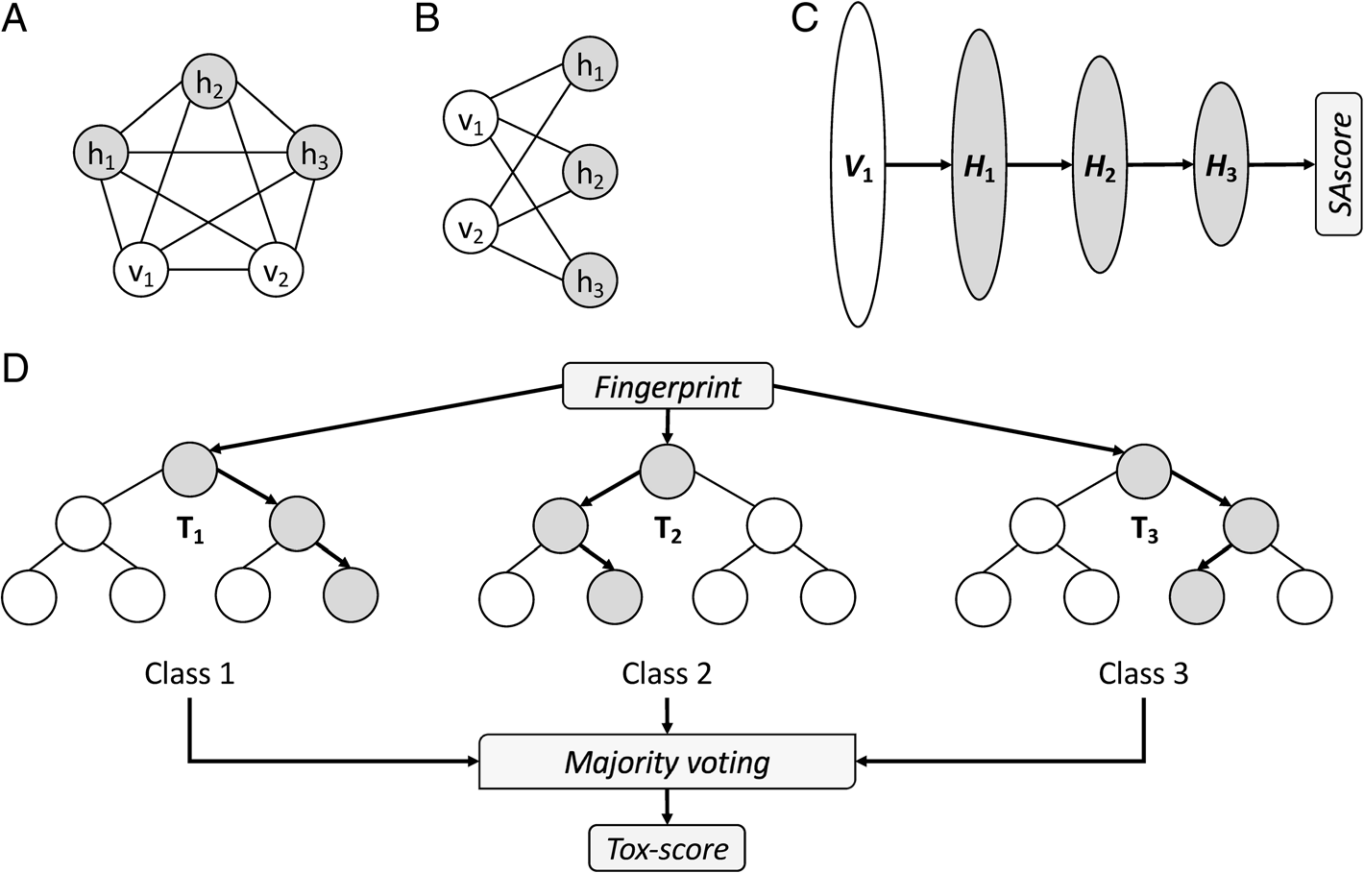
Schematics of various machine learning classifiers. (**a**) A two-layered Boltzmann Machine with 3 hidden nodes *h* and 2 visible nodes *v*. Nodes are fully connected. (**b**) A Restricted Boltzmann Machine (RBM) with the same nodes as in A. Nodes belonging to the same layer are not connected. (**c**) A Deep Belief Network with a visible layer *V* and 3 hidden layers *H*. Individual layers correspond to RBMs that are stacked against one another. (**d**) A Random Forest with 3 trees *T*. For a given instance, each tree predicts a class based on a subset of the input set. The final class assignment is obtained by the majority voting of individual trees

Numerous machine learning-based techniques have been developed to reveal complex relations between chemical entities and their biological targets [35]. In Fig. 1, we briefly present the concepts and the overall implementation of machine learning classifiers employed in this study. The first algorithm is the Restricted Boltzmann Machine (RBM), an undirected graphical model with a visible input layer and a hidden layer. In contrast to the unrestricted Boltzmann Machine, in which all nodes are connected to one another (Fig. 1A) [36], all inter-layer units in the RBM are fully connected, while there are no intra-layer connections (Fig. 1B) [37]. The RBM is an energy-based model capturing dependencies between variables by assigning an “energy” value to each configuration. The RBM is trained by balancing the probability of various regions of the state space, viz. the energy of those regions with a high probability is reduced, with the simultaneous increase in the energy of low-probability regions. The training process involves the optimization of the weight vector through Gibbs sampling [38].

The Deep Belief Network (DBN) is a generative probabilistic model built on multiple RBM units stacked against each other, where the hidden layer of an unsupervised RBM serves as the visible layer for the next sub-network (Fig. 1C) [39]. This architecture allows for a fast, layer-by-layer training, during which the contrastive divergence algorithm [40] is employed to learn a layer of features from the visible units starting from the lowest visible layer. Subsequently, the activations of previously trained features are treated as a visible unit to learn the abstractions of features in the successive hidden layer. The whole DBN is trained when the learning procedure for the final hidden layer is completed. It is noteworthy that DBNs are first effective deep learning algorithms capable of extracting a deep hierarchical representation of the training data [41].

In this study, we utilize a DBN implemented in Python with Theano and CUDA to support Graphics Processing Units (GPUs) [42]. The SAscore is predicted with a DBN architecture consisting of a visible layer corresponding to a 1024-bit Daylight fingerprint (http://www.daylight.com) and three hidden layers having 512, 128, and 32 nodes (Fig. 1C). The L2 regularization is employed to reduce the risk of overfitting. The DBN employs an adaptive learning rate decay with an initial learning rate, a decay rate, mini-batch size, the number of pre-training epochs, and the number of fine-tuning epochs of 0.01, 0.0001, 100, 20, and 1000, respectively.

Finally, the Extremely Randomized Trees, or Extra Trees (ET), algorithm [43] is used to predict the toxicity of drug candidates (Fig. 1D). Here, we employ a simpler algorithm because classification is generally less complex than regression. Classical random decision forests construct an ensemble of unpruned decision trees predicting the value of a target variable based on several input variables [44]. Briefly, a tree is trained by recursively partitioning the source set into subsets based on an attribute value test. The dataset fits well the decision tree model because each feature takes a binary value. The recursion is completed when either the subset at a node has an invariant target value or when the Gini impurity reaches a certain threshold [45]. The output class from a decision forest is simply the mode of the classes of the individual trees. The ET classifier is constructed by adding a randomized top-down splitting procedure in the tree learner. In contrast to other tree-based methods commonly employing a bootstrap replica technique, ET splits nodes by randomly choosing both attributes and cut-points, as well as it uses the whole learning sample to grow the trees. Random decision forests, including ET, are generally devoid of problems caused by overfitting to the training set because the ensemble of trees reduces model complexity leading to a classifier with a low variance. In addition, with a proper parameter tuning, the randomization procedure in ET can help achieve robust performance even for small training datasets.

The ET classifier used in this study is implemented in Python. We found empirically that the optimal performance in terms of the out-of-bag error is reached at 500 trees and adding more trees causes overfitting and increases the computational complexity. The number of features to be randomly drawn from the 1024-bit input vector is log_2_ 1024 = 10. The maximum depth of a tree is 70 with minimum numbers of 3 and 19 samples to create and split a leaf node, respectively.

### Datasets

Table 1 presents compound datasets are employed in this study. The first two sets, the Nuclei of Bioassays, Ecophysiology and Biosynthesis of Natural Products (NuBBE), and the Universal Natural Products Database (UNPD), are collections of natural products. NuBBE is a virtual database of natural products and derivatives from the Brazilian biodiversity [46], whereas UNPD is a general resource of natural products created primarily for virtual screening and network pharmacology [47]. Removing the redundancy at a Tanimoto coefficient (TC) [48] of 0.8 with the SUBSET [49] program resulted in 1008 NuBBE and 81,372 UNPD molecules. In addition to natural products, we compiled a non-redundant set of mostly synthetic bioactive compounds from the Database of Useful Decoys, Extended (DUD-E) database [50] by selecting 17,499 active molecules against 101 pharmacologically relevant targets.

**Table 1:** Compound datasets used to evaluate the performance of *e*ToxPred. These non-redundant sets are employed to train and test SAscore, Tox-score, and specific toxicities

Dataset	Size	Usage	Description
NuBBE	1008	Train/test (SAscore)	Natural products and derivatives from the Brazilian biodiversity
UNPD	81,372	Train/test (SAscore)	Diverse collection of natural products
DUD-E (actives)	17,499	Train/test (SAscore)	Mostly synthetic bioactive compounds against 102 protein targets
FDA-approved	1515	Train/test (SAscore) Train (Tox-score)	FDA approved drugs from DrugBank
KEGG-Drug	3682	Test (Tox-score)	Drugs approved in Japan, United States, and Europe
TOXNET	3035	Train (Tox-score)	Potentially hazardous chemicals
T3DB	1283	Test (Tox-score)	Collection of pollutants, pesticides, drugs, and food toxins
TCM	5883	Test (SAscore, Tox-score, unlabeled)	Traditional Chinese medicines
CP	1401	Train/test (specific toxicity)	Carcinogenic compounds tested in rodents
CD	1571	Train/test (specific toxicity)	Cardiotoxic compounds tested against hERG potassium channel
ED	17,059	Train/test (specific toxicity)	Endocrine disrupting compounds tested against androgen and estrogen receptors
AO	12,612	Train/test (specific toxicity)	Toxins from various sources annotated with acute oral toxicity

The next two sets, FDA-approved and Kyoto Encyclopedia of Genes and Genomes (KEGG) Drug, comprise molecules approved by regulatory agencies, which possess acceptable risk versus benefit ratios. Although these molecules may still cause adverse drug reactions, we refer to them as non-toxic because of their relatively high therapeutic indices. FDA-approved drugs were obtained from the DrugBank database, a widely used cheminformatics resource providing comprehensive information on known drugs and their molecular targets [51]. The KEGG-Drug resource contains drugs approved in Japan, United States, and Europe, annotated with the information on their targets, metabolizing enzymes, and molecular interactions [52]. Removing the chemical redundancy from both datasets yielded 1515 FDA-approved and 3682 KEGG-Drug compounds.

Two counter-datasets, TOXNET and the Toxin and Toxin Target Database (T3DB), contain compounds indicated to be toxic. The former resource maintained by the National Library of Medicine provides databases on toxicology, hazardous chemicals, environmental health, and toxic releases [53]. Here, we use the Hazardous Substances Data Bank focusing on the toxicology of potentially hazardous chemicals. T3DB houses detailed toxicity data in terms of chemical properties, molecular and cellular interactions, and medical information, for a number of pollutants, pesticides, drugs, and food toxins [54]. These data are extracted from multiple sources including other databases, government documents, books, and scientific literature. The non-redundant sets of TOXNET and T3DB contain 3035 and 1283 toxic compounds, respectively.

As an independent set, we employ the Traditional Chinese Medicine (TCM) Database@Taiwan, currently the largest and most comprehensive small molecule database on traditional Chinese medicine for virtual screening [55]. TCM is based on information collected from Chinese medical texts and scientific publications for 453 different herbs, animal products, and minerals. From the original dataset, we first selected molecules with a molecular weight in the range of 100–600 Da, and then removed redundancy at a TC of 0.8, producing a set of 5883 unique TCM compounds.

Finally, we use four datasets to evaluate the prediction of specific toxicities. Compounds causing cancer in high dose tests were obtained from the Carcinogenicity Potency (CP) database [56]. These data are labeled based on series of experiments carried out on rodents considering different tissues of the subjects. A chemical is deemed toxic if it caused tumor growth in at least one tissue specific experiment. The CP set comprises 796 toxic and 605 non-toxic compounds. The cardiotoxicity (CD) dataset contains 1571 molecules characterized with bioassay against human ether-a-go-go related gene (hERG) potassium channel. hERG channel blockade induces lethal arrhythmia causing a life-threatening symptom [57]. The CD set includes 350 toxic compounds with an IC_50_ of < 1 μm [58]. The endocrine disruption (ED) dataset is prepared based on the bioassay data for androgen and estrogen receptors taken from the Tox21 Data Challenge. Endocrine disrupting chemicals interfere with the normal functions of endogenous hormones causing metabolic and reproductive disorders, the dysfunction of neuronal and immune systems, and cancer growth [59]. The ED set contains 1317 toxic and 15,742 non-toxic compounds. The last specific dataset is focused on the acute oral toxicity (AO). Among 12,612 molecules with LD_50_ data provided by the SuperToxic database [60], 7392 compounds are labeled as toxic with a LD_50_ of < 500 mg kg^− 1^. It is important to note that since LD_50_ is not indicative of non-lethal toxic effects, a chemical with a high LD_50_ may still cause adverse reactions at small doses.

### Model training, cross-validation, and evaluation

Input data to machine learning models are 1024-bit Daylight fingerprints constructed for dataset compounds with Open Babel [61]. The reference SAscore values are computed with an exact approach that combines the fragment-based score representing the “historical synthetic knowledge” with the complexity-based score penalizing the presence of ring systems, such as spiro and fused rings, multiple stereo centers, and macrocycles [62]. The DBN-based predictor of the SAscore was trained and cross-validated against NuBBE, UNPD, FDA-approved, and DUD-E-active datasets. Cross-validation is a common technique used in statistical learning to evaluate the generalization of a trained model [63]. In a *k*-fold cross-validation protocol, one first divides the dataset into *k* different subsets and then the first subset is used as a validation set for a model trained on the remaining *k* – 1 subsets. This procedure is repeated *k* times employing different subsets as the validation set. Averaging the performance obtained for all *k* subsets yields the overall performance and estimates the validation error of the model. In this work, the SAscore predictor is evaluated with a 5-fold cross-validation protocol, which was empirically demonstrated to be sufficient for most applications [64].

The Tox-score prediction is conducted with a binary, ET-based classifier. The training and cross-validation are carried out for the FDA-approved dataset used as positive (non-toxic) instances and the TOXNET dataset used as negative (toxic) instances. Subsequently, the toxicity predictor is trained on the entire FDA-approved/TOXNET dataset and then independently tested against the KEGG-Drug (positive, non-toxic) and T3DB (negative, toxic) sets. In addition, the capability of the classifier to predict specific toxicities is assessed against CP, CD, ED, and AO datasets. Similar to the SAscore predictor, a 5-fold cross-validation protocol is employed to rigorously evaluate the performance of the toxicity classifier. Finally, both machine learning predictors of SAscore and Tox-score are applied to the TCM dataset.

The performance of *e*ToxPred is assessed with several metrics derived from the confusion matrix, the accuracy (ACC), the sensitivity or true positive rate (TPR), and the fall-out or false positive rate (FPR):

1 $$ \mathrm{ACC}=\frac{TP+ TN}{TP+ FP+ TN+ FN} $$

2 $$ \mathrm{TPR}=\frac{TP}{TP+ FN} $$

3 $$ \mathrm{FPR}=\frac{FP}{FP+ TN} $$

where *TP* is the number of true positives. i.e. non-toxic compounds classified as non-toxic, and *TN* is the number of true negatives, i.e. toxic compounds classified as toxic. *FP* and *FN* are the numbers of over- and under-predicted non-toxic molecules, respectively.

In addition, we assess the overall quality of a binary classifier with the Matthews correlation coefficient (MCC) [65] and the Receiver Operating Characteristic (ROC) analysis. The MCC is generally regarded as a well-balanced measure ranging from − 1 (anti-correlation) to 1 (a perfect classifier) with values around 0 corresponding to a random guess:

4 $$ \mathrm{MCC}=\frac{TN\times TP- FP\times FN}{\sqrt{\left( TP+ FP\right)\left( TP+ FN\right)\left( TN+ FP\right)\left( TN+ FN\right)}} $$

where *TP*, *TN*, *FP*, and *FN* are defined above. The ROC analysis describes a trade-off between the FPR and the TPR for a classifier at varying decision threshold values. The MCC and ROC are important metrics to help select the best model considering the cost and the class distribution. The hyperparameters of the model, including the number of features resulting in the best split, the minimum number of samples required to split an internal node, and the minimum number of samples required to be at a leaf node, are tuned with a grid search method. The best set of hyperparameters maximizes both the MCC and ROC.

Finally, the performance of the regression classifier is evaluated with the mean squared error (MSE) and the Pearson correlation coefficient (PCC) [66]. The MSE is a risk function measuring the average of the squares of the errors:

5 $$ \mathrm{MSE}=\frac{1}{N}\sum \limits_{i=1}^N{\left(\widehat{y_i}-{y}_i\right)}^2 $$

where *N* is the total number of evaluation instances, and $\widehat{y_i}$ and *y*_*i*_ are the predicted and actual values of *i*-th instance, respectively. Further, the PCC is often employed to assess the accuracy of point estimators by measuring the linear correlation between the predicted and actual values. Similar to the MCC, PCC ranges from − 1 to 1, where − 1 is a perfect anti-correlation, 1 is a perfect correlation, and 0 is the lack of any correlation. It is calculated as:

6 $$ \mathrm{PCC}=\frac{\operatorname{cov}\left(\widehat{y},y\right)}{\sigma_{\widehat{y}}{\sigma}_y} $$

where $\operatorname{cov}\left(\widehat{y},y\right)$ is the covariance matrix of the predicted and actual values, and ${\sigma}_{\widehat{y}}$ and *σ*_*y*_ are the standard deviations of the predicted and actual values, respectively.

## Results and discussion

### SAscore prediction with eToxPred

**Fig. 2:**
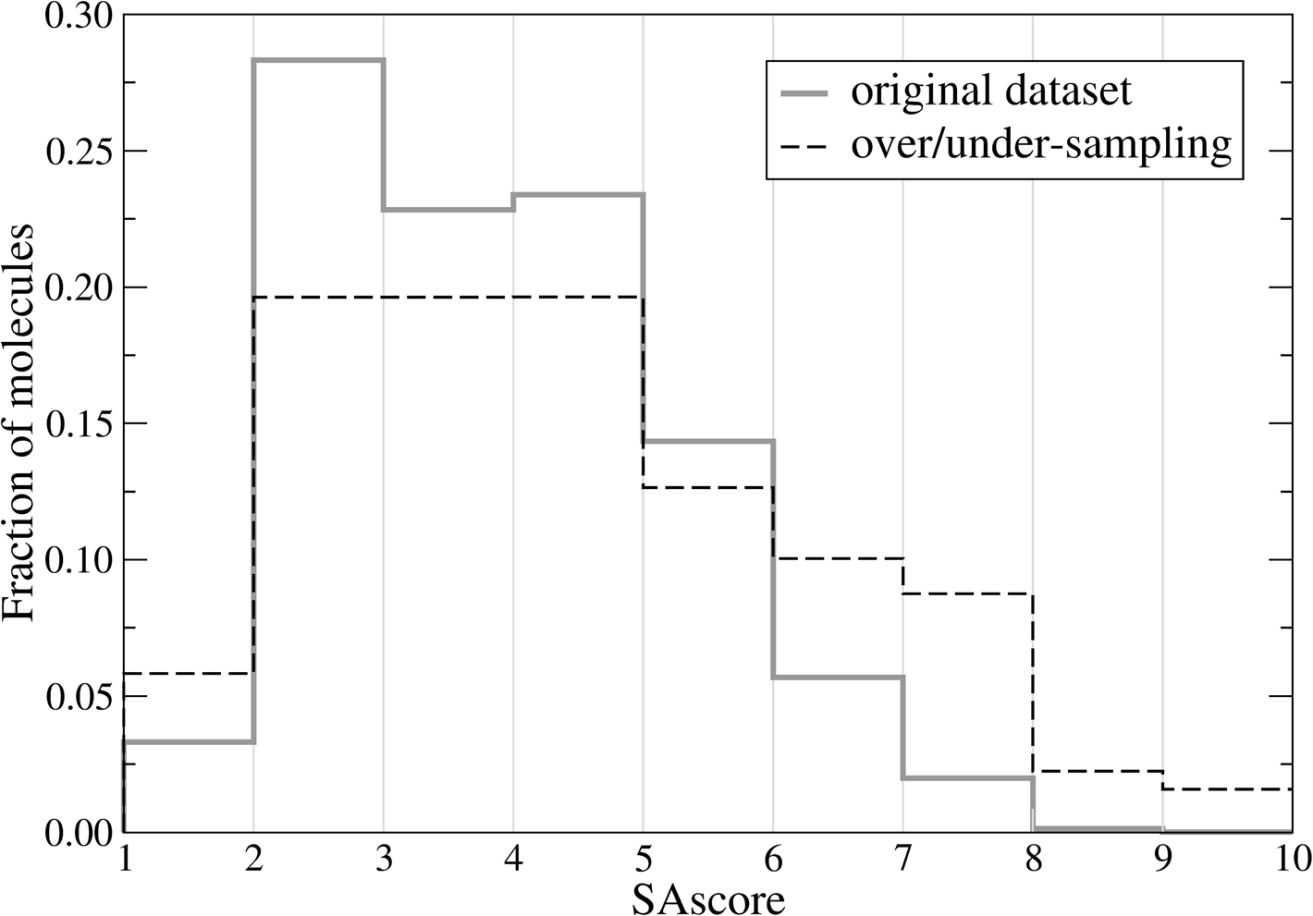
Resampling strategy to balance the dataset. The histogram shows the distribution of SAscore values across the training set before (solid gray line) and after (dashed black line) the over/under-sampling

The SAscore combining contributions from various molecular fragments and a complexity penalty, was developed to help estimate the synthetic accessibility of organic compounds [62]. It ranges from 1 for molecules easy to make, up to 10 for those compounds that are very difficult to synthetize. The datasets used to train and validate the SAscore predictor, including FDA-approved, DUD-E-active, NuBBE, and UNPD datasets, are highly skewed, i.e., SAscore values are non-uniformly distributed over the 1–10 range. For instance, Fig. 2 (solid gray line) shows that as many as 28.3% of molecules in the original dataset have a SAscore between 2 and 3. Therefore, a pre-processing is needed to balance the dataset for a better performance of the SAscore predictor. Specifically, an over/under-sampling procedure is employed by duplicating those cases with under-represented SAscore values and randomly selecting a subset of over-represented instances. The over-sample ratio for the 1–2 range is 2. The number of data points in the 2–5 range are uniformly under-sampled to 90,000, whereas those in the 5–6 range remain unchanged. For 6–7, 7–8, 8–9, and 9–10 ranges, the over-sample ratios are 2, 5, 20, and 100, respectively. Figure 2 (dashed black line) shows that the over/under-sampled set contains more instances with low (1–2) and high (6–10) SAscore values compared to the original dataset.

**Fig. 3:**
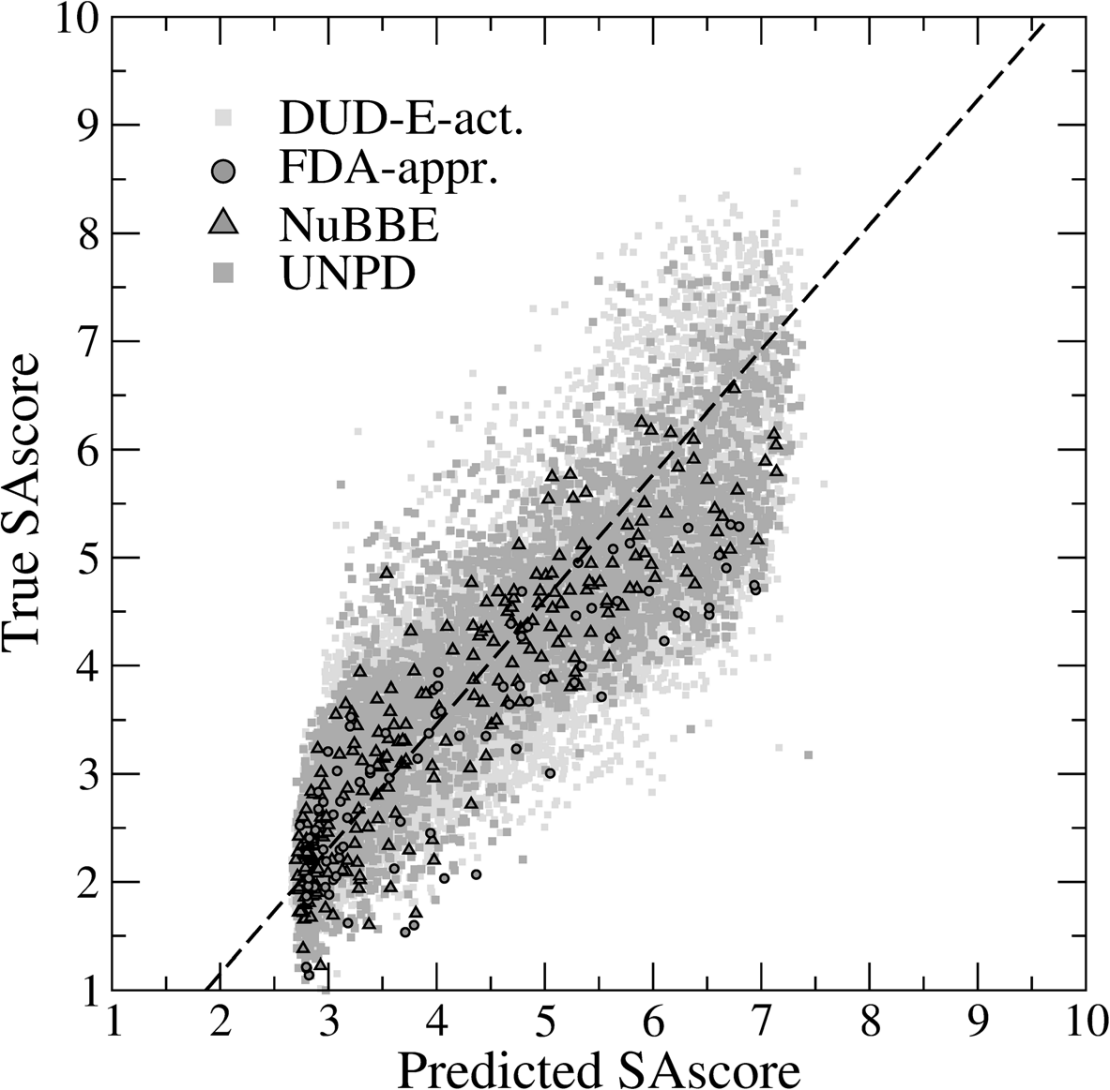
SAscore prediction for several datasets. The scatter plot shows the correlation between the predicted and true SAscore values for active compounds from the Directory of Useful Decoys, Extended (DUD-E), FDA-approved drugs, and natural products from the NuBBE and UNPD databases. The regression line is dashed black

**Fig. 4:**
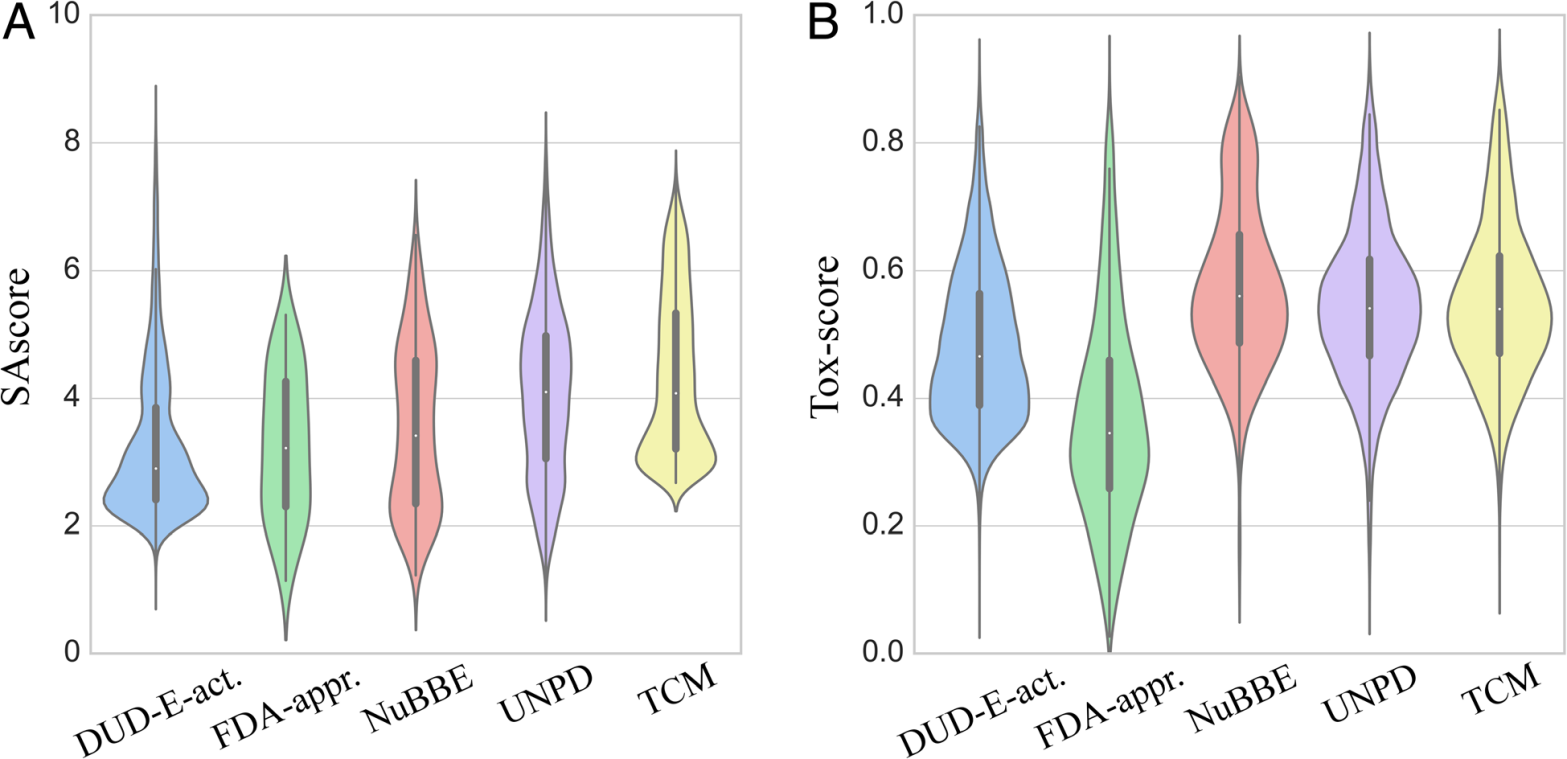
SAscore and Tox-score prediction for several datasets. Violin plots show the distribution of (**a**) SAscore and (**b**) Tox-score values across active compounds from the Directory of Useful Decoys, Extended (DUD-E), FDA-approved drugs, natural products from the NuBBE and UNPD databases, and traditional Chinese medicines (TCM)

A scatter plot of the predicted vs. actual SAscore values is shown in Fig. 3 for FDA-approved, DUD-E-active, NuBBE, and UNPD datasets. Encouragingly, the cross-validated PCC (Eq. 6) across all four datasets is as high as 0.89 with a low MSE (Eq. 5) of 0.81 (~ 4%) for the predicted SAscore. Next, we apply the DBN predictor to individual datasets and analyze the distribution of the estimated SAscore values in Fig. 4. As expected, mostly synthetic molecules from the DUD-E-active dataset have the lowest median SAscore of 2.9, which is in line with values previously reported for catalogue and bioactive molecules from the World Drug Index (http://www.daylight.com/products/wdi.html) and MDL Drug Data Report (http://www.akosgmbh.de/accelrys/databases/mddr.htm) databases. The median SAscore for FDA-approved drugs is 3.2 because in addition to synthetic and semi-synthetic compounds, this heterogeneous dataset also contains natural products whose chemical structures are generally more complex than the “standard” organic molecules. Both datasets of natural products, NuBBE and UNPD, have even higher median SAscore values of 3.4 and 4.1, respectively. Further, similar to the analysis of the Dictionary of Natural Products (http://dnp.chemnetbase.com) conducted previously [62], natural products employed in the present study have a characteristic bimodal distribution with two distinct peaks at a SAscore of about 3 and 5. Finally, the median SAscore for TCM is 4.1 concurring with those values calculated for natural products. Interestingly, a number of TCM molecules have relatively high synthetic accessibility and the shape of the distribution of the estimated SAscore values is similar to that for the active compounds from the DUD-E dataset. Overall, the developed DBN-based model is demonstrated to be highly effective in estimating the SAscore directly from binary molecular fingerprints.

### Tox-score prediction with eToxPred

**Fig. 5:**
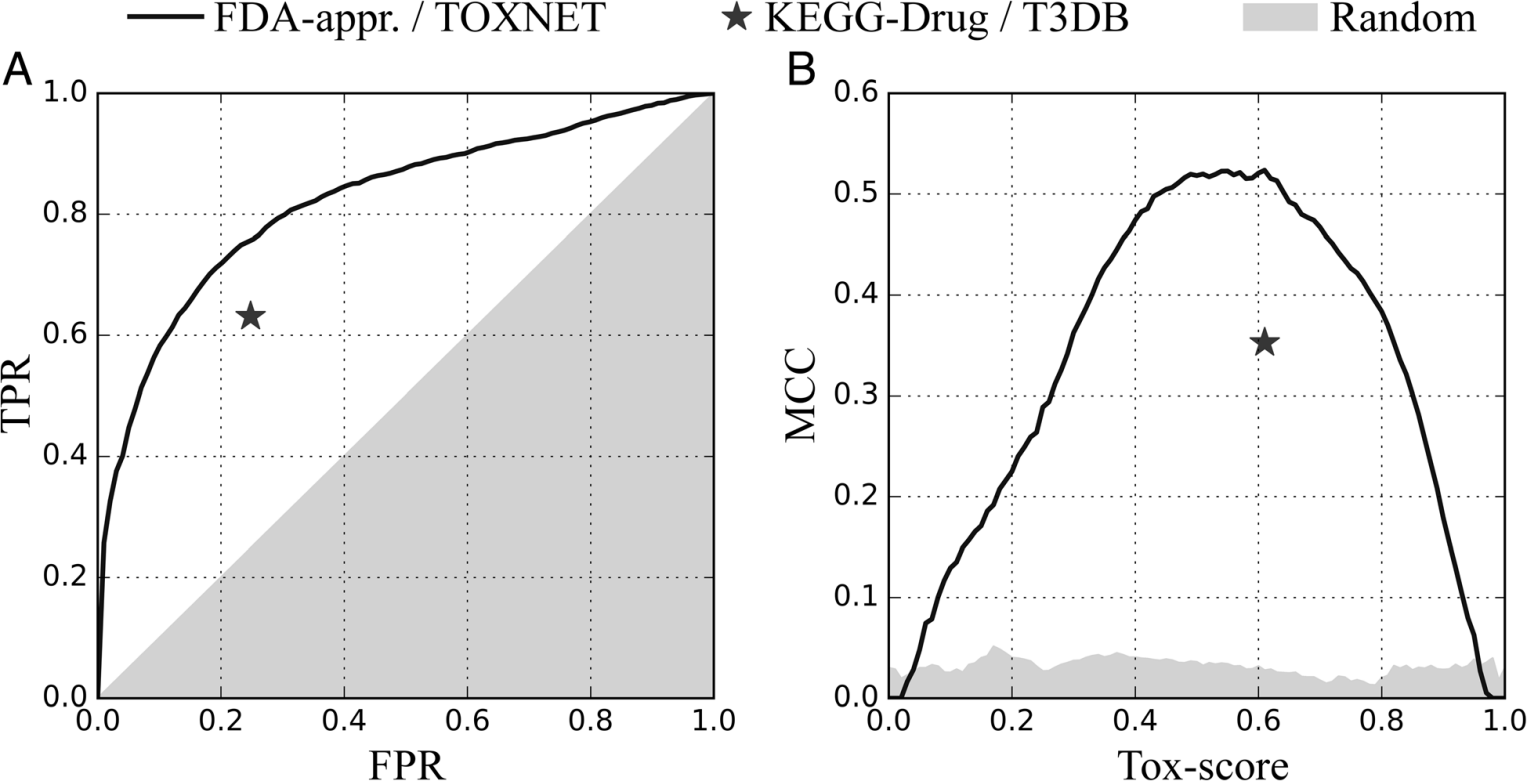
Performance of *e*ToxPred in the prediction of toxic molecules. (**a**) The receiver operating characteristic plot and (**b**) the Matthews correlation coefficient (MCC) plotted as a function of the varying Tox-score. TPR and FPR are the true and false positive rates, respectively. Gray areas correspond to the performance of a random classifier. *e*ToxPred is first applied to the primary training set (FDA-approved / TOXNET, solid black lines) to select the optimum Tox-score threshold. Then, the optimized *e*ToxPred is applied to the independent testing set (KEGG-Drug and T3DB, solid black stars)

*e*ToxPred was developed to quickly estimate the toxicity of large collections of low molecular weight organic compounds. It employs an ET classifier to compute the Tox-score ranging from 0 (a low probability to be toxic) to 1 (a high probability to be toxic). The primary dataset to evaluate *e*ToxPred consists of FDA-approved drugs, considered to be non-toxic, and potentially hazardous chemicals from the TOXNET database. Figure 5 shows the cross-validated performance of *e*ToxPred in the prediction of toxic molecules. The ROC curve in Fig. 5A demonstrates that the ET classifier is highly accurate with the area under the curve (AUC) of 0.82. According to Fig. 5B, a Tox-score of 0.58 the most effectively discriminates between toxic and non-toxic molecules, yielding an MCC (Eq. 4) of 0.52. Employing this threshold gives a high TPR of 0.71 at a low FPR of 0.19.

Next, we apply *e*ToxPred with the optimized Tox-score threshold to an independent dataset consisting of KEGG-Drug molecules, regarded as non-toxic, and toxic substances obtained from T3DB. Despite the fact that many of these compounds are unseen to the ET classifier, *e*ToxPred quite efficiently recognizes toxic molecules. The MCC for the KEGG-Drug and T3DB datasets is 0.35, corresponding to the TPR and FPR of 0.63 and 0.25, respectively. Table 2 shows that using the ET classifier yields the best performance on this independent dataset compared to other machine learning techniques. Even though RF is slightly more accurate than ET against FDA-approved and TOXNET, the performance of ET is noticeably higher for KEGG-Drug and T3DB. In addition, we tested two other classifiers, the Linear Discriminant Analysis (LDA) [67] and Multilayer Perceptron (MLP) [68], however, their performance is generally not as high as those of RF and ET. Furthermore, the results obtained for the TCM dataset show that ET has the lowest tendency to over-predict the toxicity compared to other classifiers (the last row in Table 2).

**Table 2:** Performance of various machine learning classifiers to predict toxicity. The following classifiers are tested

Dataset	Metric	Toxicity classifiers			
		LDA	MLP	RF	ET
FDA-appr. / TOXNET	ACC	0.745	0.744	**0.760**	0.756
	TPR / FPR	0.723 / 0.232	0.679 / 0.180	0.733 / 0.218	0.719 / 0.186
	MCC	0.495	0.525	**0.528**	0.523
KEGG-Drug / T3DB	ACC	0.647	0.645	0.674	**0.721**
	TPR / FPR	0.671 / 0.362	0.675 / 0.365	0.688 / 0.331	0.631 / 0.248
	MCC	0.272	0.273	0.316	**0.353**
TCM	Tox-score	0.504 ± 0.013	0.537 ± 0.242	0.574 ± 0.143	0.552 ± 0.122
	% toxic	63.9	61.8	68.5	59.7

Linear Discriminant Analysis (LDA), Multi-Layer Perceptron (MLP), Random Forest (RF), and Extra Trees (ET). Individual models are first trained and 5-fold cross-validated against FDA-approved and TOXNET datasets and then applied to KEGG-Drug and T3DB as an additional validation against independent datasets. The performance of toxicity classifiers on FDA-approved / TOXNET and KEGG-Drug / T3DB datasets is assessed with the accuracy (ACC, Eq. 1), true (TPR, Eq. 2) and false (FPR, Eq. 3) positive rates, and the Matthews correlation coefficient (MCC, Eq. 4). The best performance across all models in terms of the highest ACC and MCC values are highlighted in bold. Finally, the trained models are applied to estimate the toxicity of traditional Chinese medicines in the TCM dataset and the average ± standard deviation Tox-score values as well as the percentage of predicted toxic molecules are reported

**Fig. 6:**
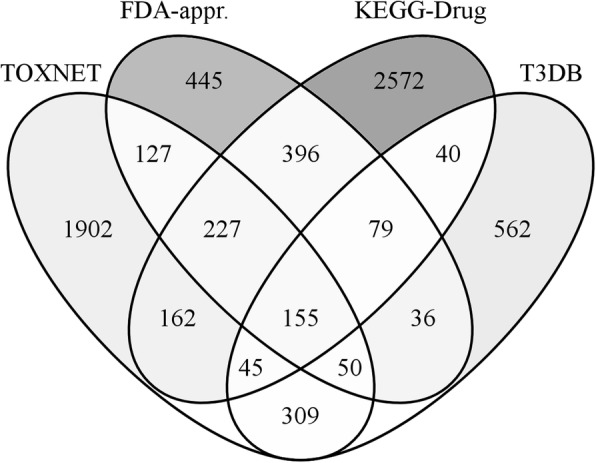
Venn diagrams showing the overlap among various datasets. FDA-approved and TOXNET are the primary training datasets, whereas KEGG-Drug and T3DB are independent testing sets

Switching to an independent dataset causes the performance of machine learning classifiers to deteriorate on account of a fair amount of ambiguity in the training and testing sets. To better understand the datasets, we present a Venn diagram in Fig. 6. For instance, FDA-approved and TOXNET share as many as 559 molecules, whereas the intersection of KEGG-Drug and T3DB consists of 319 compounds. Further, 36 molecules classified as non-toxic in the FDA-approved / TOXNET dataset are labelled toxic in the KEGG-Drug / T3DB dataset (162 compounds are classified the other way around). As a result, the accuracy of both LDA and MLP drops from 0.74 to 0.65, however, the accuracy of ET only slightly decreases from 0.76 to 0.72, demonstrating the robustness of this classifier. Indeed, ET was previously shown to be resilient to high-noise conditions [43], therefore, we decided to employ this machine learning technique as a default classifier in *e*ToxPred.

We also apply *e*ToxPred to evaluate the compound toxicity across several datasets used to predict the synthetic accessibility. Not surprisingly, Fig. 4B shows that FDA-approved drugs have the lowest median Tox-score of 0.34. The toxicity of active compounds from the DUD-E dataset is a bit higher with a median Tox-score of 0.46. Molecules in both natural products datasets as well as traditional Chinese medicines are assigned even higher toxicity values; the median Tox-score is 0.56, 0.54, and 0.54 for NuBBE, UNPD, and TCM, respectively. These results are in line with other studies examining the composition and toxicology of TCM, for instance, toxic constituents from various TCM sources include alkaloids, glycosides, peptides, amino acids, phenols, organic acids, terpenes, and lactones [69].

**Fig. 7:**
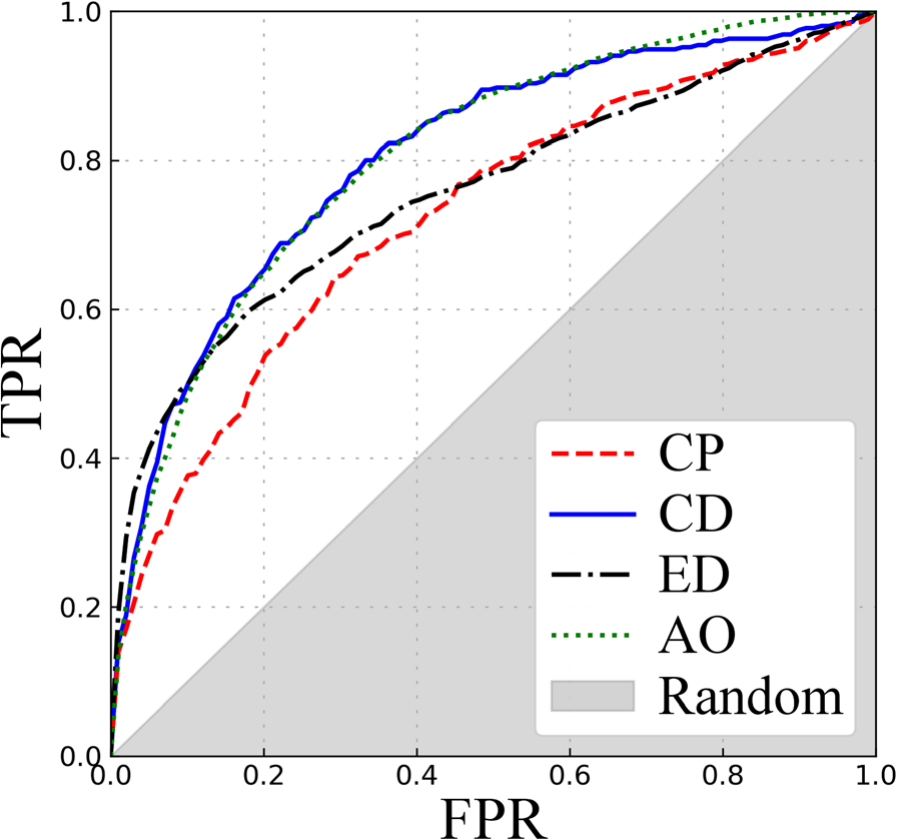
Performance of *e*ToxPred in the prediction of specific toxicities. The receiver operating characteristic plots are shown for Carcinogenicity Potency (CP), cardiotoxicity (CD), endocrine disruption (ED), and acute oral toxicity (AO)

Finally, the prediction of specific toxicities is assessed against four independent datasets. Figure 7 and Table 3 show that the performance of *e*ToxPred is the highest against the AO and CD datasets with AUC values of 0.80. The performance against the remaining datasets, CP (AUC of 0.72) and ED (AUC of 0.75), is only slightly lower. These results are in line with benchmarking data reported for other classifiers; for instance, *e*ToxPred compares favorably with different methods particularly against the AO and ED datasets [30, 70]. Importantly, the ET-based classifier employing molecular fingerprints turns out to be highly effective predicting not only the general toxicity, but also specific toxicities as demonstrated for the carcinogenicity potency, cardiotoxicity, endocrine disruption, and acute oral toxicity.

**Table 3:** Performance of the Extra Trees classifier to predict specific toxicities

Dataset	AUC	ACC
CP	0.721	0.722
CD	0.799	0.798
ED	0.750	0.744
AO	0.800	0.854

The following datasets are used: carcinogenicity potency (CP), cardiotoxicity (CD), endocrine disruption (ED), and acute oral toxicity (AO). The performance is assessed with the area under the curve (AUC) and the accuracy (ACC, Eq. 1)

### Composition of non-toxic compounds

**Fig. 8:**
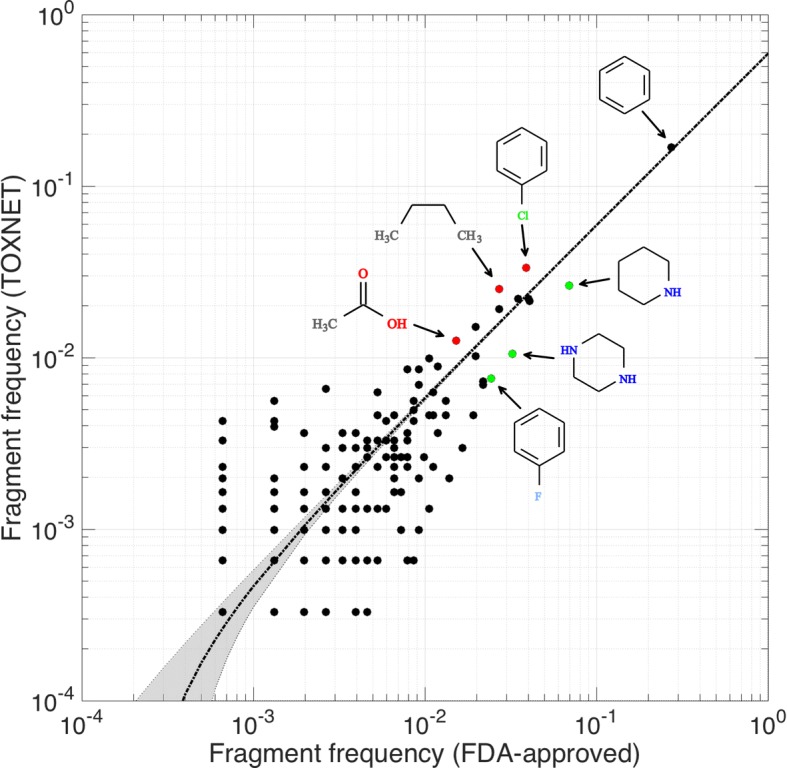
Composition of non-toxic and toxic compounds. The scatter plot compares the frequencies of chemical fragments extracted with *e*MolFrag from FDA-approved (non-toxic) and TOXNET (toxic) molecules. The regression line is dotted black and the gray area delineates the corresponding confidence intervals. Three selected examples of fragments more commonly found in FDA-approved molecules (piperidine, piperazine, and fluorophenyl) are colored in green, whereas three counter examples of fragments more frequent in the TOXNET dataset (chlorophenyl, *n*-butyl, and acetic acid) are colored in red

**Fig. 9:**
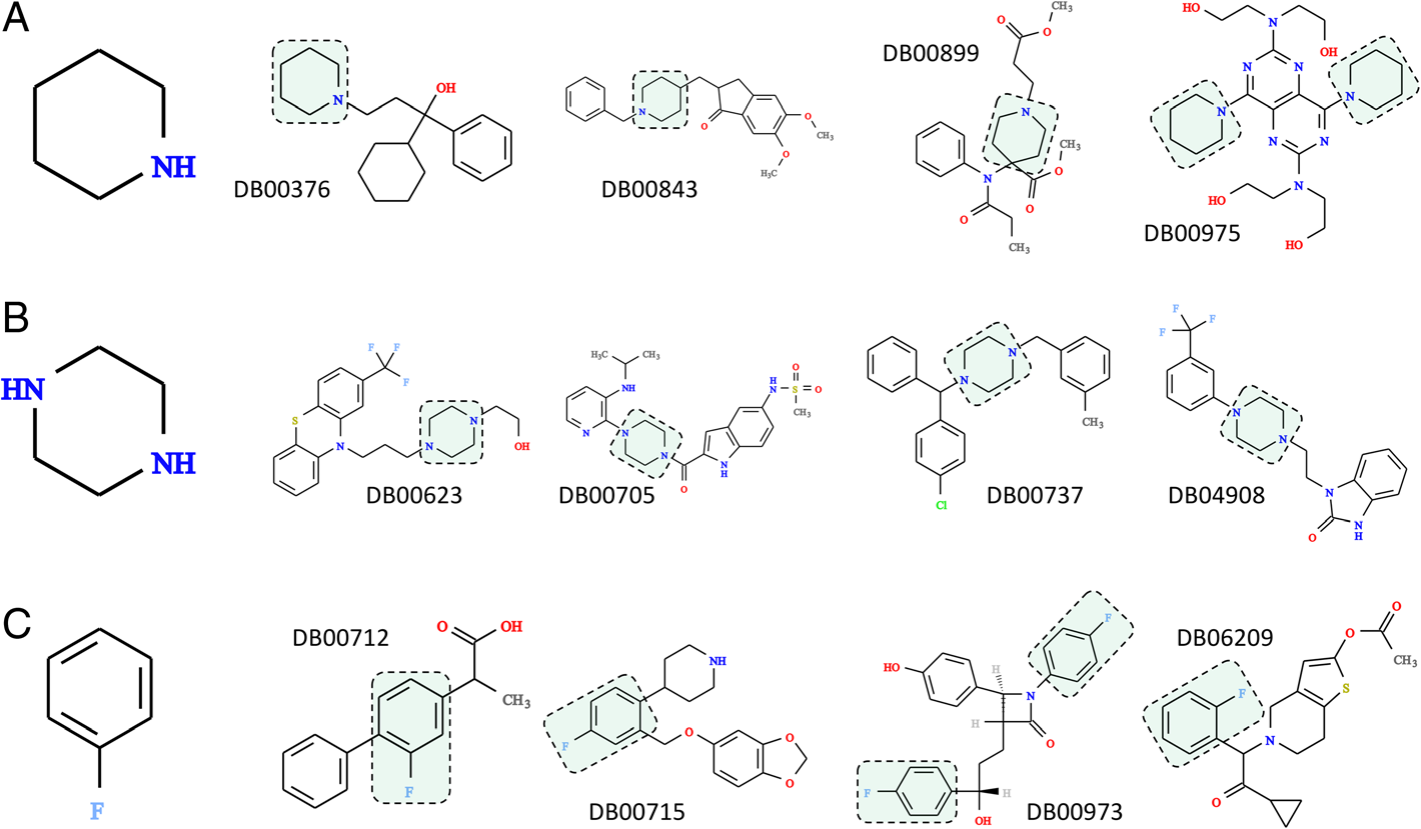
Composition of selected non-toxic compounds. Three examples of fragments more commonly found in FDA-approved molecules than in the TOXNET dataset: (**a**) piperidine, (**b**) piperazine, and (**c**) fluorophenyl. Four sample molecules containing a particular moiety (highlighted by green boxes) are selected from DrugBank and labeled by the DrugBank-ID

**Fig. 10:**
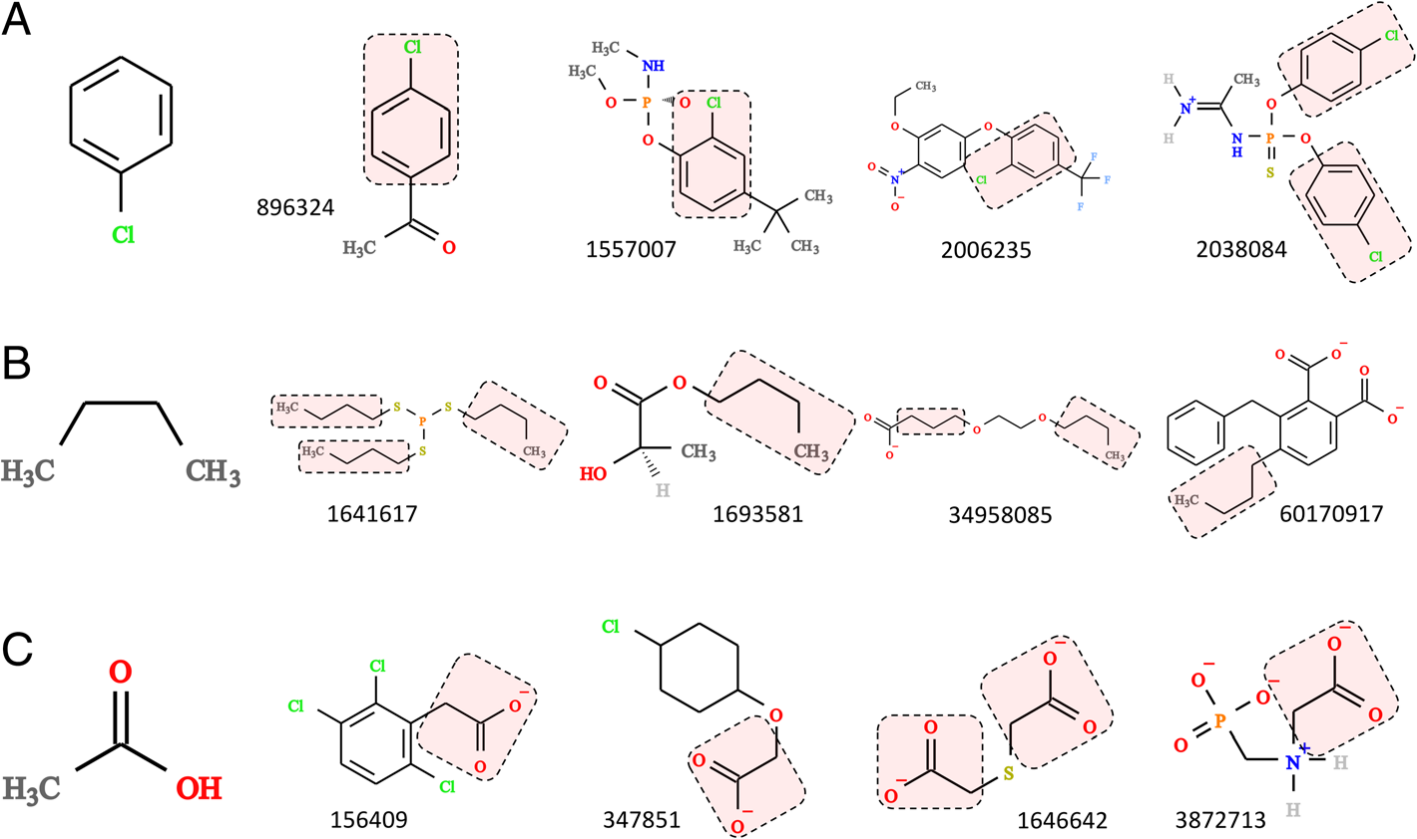
Composition of selected toxic compounds. Three examples of fragments more commonly found in the TOXNET dataset than in FDA-approved molecules: (**a**) chlorophenyl, (**b**) *n*-butyl, and (**c**) acetic acid. Four sample molecules containing a particular moiety (highlighted by red boxes) are selected from ZINC and labeled by the ZINC-ID

Since *e*ToxPred quite effectively estimates the toxicity of small organic compounds from their molecular fingerprints, there should be some discernible structural attributes of toxic and non-toxic substances. On that account, we decomposed FDA-approved and TOXNET molecules into chemical fragments with *e*MolFrag [9] in order to compare their frequencies in both datasets. Figure 8 shows a scatter plot of 698 distinct fragments extracted by *e*MolFrag. As expected, the most common moiety is a benzene ring, whose frequency is 0.27 in the FDA-approved and 0.17 in TOXNET fragment sets. In general, fragment frequencies are highly correlated with a PCC of 0.98, however, certain fragments are more often found in either dataset. To further investigate these cases, we selected three examples of fragments more commonly found in FDA-approved molecules, represented by green dots below the regression line in Fig. 8, and three counter examples of those fragments that are more frequent in the TOXNET dataset, shown as red dots above the regression line in Fig. 8. In addition, the selected parent molecules for these fragments are presented in Fig. 9 (FDA-approved) and Fig. 10 (TOXNET).

Examples shown in Fig. 9 include piperidine (Fig. 9A), piperazine (Fig. 9B), and fluorophenyl (Fig. 9C) moieties, whose frequencies in FDA-approved/TOXNET datasets are 0.069/0.026, 0.032/0.010, and 0.024/0.007, respectively. Nitrogen-bearing heterocycles, piperidine and piperazine, are of central importance to medicinal chemistry [71]. Piperidine offers a number of important functionalities that have been exploited to develop central nervous system modulators, anticoagulants, antihistamines, anticancer agents and analgesics [72]. This scaffold is the basis for over 70 drugs, including those shown in Fig. 9A, trihexyphenidyl (DrugBank-ID: DB00376), a muscarinic antagonist to treat Parkinson’s disease [73], donepezil (DrugBank-ID: DB00843), a reversible acetyl cholinesterase inhibitor to treat Alzheimer’s disease [74], an opioid analgesic drug remifentanil (DrugBank-ID: DB00899) [75], and dipyridamole (DrugBank-ID: DB00975), a phosphodiesterase inhibitor preventing the blood clot formation [76].

Similarly, many well established and commercially available drugs contain a piperazine ring as part of their molecular structures [77]. A wide array of pharmacological activities exhibited by piperazine derivatives make them attractive leads to develop new antidepressant, anticancer, anthelmintic, antibacterial, antifungal, antimalarial, and anticonvulsant therapeutics [78]. Selected examples of piperazine-based drugs presented in Fig. 9B, are antipsychotic fluphenazine (DrugBank-ID: DB00623), antiretroviral delavirdine (DrugBank-ID: DB00705), antihistamine meclizine (DrugBank-ID: DB00737), and flibanserin (DrugBank-ID: DB04908) to treat hypoactive sexual desire disorder among pre-menopausal women [79]. All of these compounds contain substituents at both N1- and N4-positions, which concurs with the analysis of piperazine substitution patterns across FDA-approved pharmaceuticals revealing that 83% of piperazine-containing drugs are substituted at both nitrogens, whereas only a handful have a substituent at any other position [77].

Incorporating fluorine into drug leads is an established practice in drug design and optimization. In fact, so-called fluorine scan is often employed in the development of drug candidates to systematically exploit the benefits of fluorine substitution [80]. As a result, an estimated one-third of the top-performing drugs currently on the market contain fluorine atoms in their structure [81]. The presence of fluorine atoms in pharmaceuticals increases their bioavailability by modulating p*K*_a_ and lipophilicity, as well as by improving their absorption and partitioning into membranes [82]. Further, fluorination helps stabilize the binding of a drug to a protein pocket by creating additional favorable interactions, as it was suggested for the fluorophenyl ring of paroxetine (DrugBank-ID: DB00715) [83], a selective serotonin reuptake inhibitor shown in Fig. 9C. A low metabolic stability due to cytochrome P450-mediated oxidation can be mitigated by blocking metabolically unstable hydrogen positions with fluorine atoms [84], as exemplified by drug structures shown in Fig. 9C. Indeed, a targeted fluorination of a nonsteroidal anti-inflammatory drug flurbiprofen (DrugBank-ID: DB00712) helped prolong its metabolic half-life [85]. Another example is cholesterol inhibitor ezetimibe (DrugBank-ID: DB00973), in which two metabolically labile sites are effectively blocked by fluorine substituents [86]. Finally, replacing the chlorine atom with a fluorine improves safety profile and pharmacokinetic properties of prasugrel (DrugBank-ID: DB06209) compared to other thienopyridine antiplatelet drugs, ticlopidine and clopidogrel [87].

### Composition of toxic compounds

Next, we selected three counter examples (red dots in Fig. 8) of fragments frequently found in toxic substances, chlorophenyl, *n*-butyl, and acetic acid, whose representative parent molecules are presented in Fig. 10. For instance, the chlorophenyl moiety (Fig. 10A) is the constituent of *p*-chloroacetophenone (ZINC-ID: 896324) used as a tear gas for riot control, crufomate (ZINC-ID: 1557007), an insecticide potentially toxic to humans, the herbicide oxyfluorfen (ZINC-ID: 2006235), and phosacetim (ZINC-ID: 2038084), a toxic acetylcholinesterase inhibitor used as a rodenticide. Further, *n*-butyl groups (Fig. 10B) are present in a number of toxic substances, including merphos (ZINC-ID: 1641617), a pesticide producing a delayed neurotoxicity in animals, *n*-butyl lactate (ZINC-ID: 1693581), an industrial chemical and food additive, diethylene glycol monobutyl ether acetate (ZINC-ID: 34958085) used as solvents for cleaning fluids, paints, coatings and inks, and *n*-butyl benzyl phthalate (ZINC-ID: 60170917), a plasticizer for vinyl foams classified as toxic in Europe and excluded from the manufacturing of toys and child care products in Canada. The last example is the acetic acid moiety (Fig. 10C) found in many herbicides, e.g. chlorfenac (ZINC-ID: 156409), 4-chlorophenoxyacetic acid (ZINC-ID: 347851), and glyphosate (ZINC-ID: 3872713) as well as in thiodiacetic acid (ZINC-ID: 1646642), a chemical used by the material industry to synthesize sulfur-based electro-conductive polymers.

## Conclusions

In this study, we developed a new program to predict the synthetic accessibility and toxicity of small organic compounds directly from their molecular fingerprints. The estimated toxicity is reported as the Tox-score, a new machine learning-based scoring metric implemented in *e*ToxPred, whereas the synthetic accessibility is evaluated with the SAscore, an already established measure in this field. We previously developed tools, such as *e*MolFrag and *e*Synth, to build large, yet target-specific compound libraries for virtual screening. *e*ToxPred can be employed as a post-generation filtering step to eliminate molecules that are either difficult to synthesize or resemble toxic substances included in TOXNET and T3DB rather than FDA-approved drugs and compounds listed by the KEGG-Drug dataset. Additionally, it effectively predicts specific toxicities, such as the carcinogenicity potency, cardiotoxicity, endocrine disruption, and acute oral toxicity. In principle, this procedure could save considerable resources by concentrating the subsequent virtual screening and molecular modeling simulations on those compounds having a better potential to become leads.

### Availability and requirements

***Project name:*** *e*ToxPred.

***Project home page:*** https://github.com/pulimeng/etoxpred

***Operating system(s):*** Platform independent.

***Programming language:*** Python 2.7+ or Python 3.5+.

***Other requirements:*** Theano, numpy 1.8.2 or higher, scipy 0.13.3 or higher, scikit-learn 0.18.1, OpenBabel 2.3.1, CUDA 8.0 or higher (optional).

***License:*** GNU GPL.

***Any restrictions to use by non-academics:*** license needed.

## Abbreviations

ACC: accuracy
ADMET: absorption, distribution, metabolism, excretion, and toxicity
CADD: computer-aided drug discovery
DBN: deep belief network
DNN: deep neural network
DUD-E: Database of Useful Decoys, Extended
ET: extra trees
FDA: Food and Drug Administration
FPR: false positive rate
GPU: graphics processing units
hERG: human Ether-à-go-go-Related Gene
KEGG: Kyoto Encyclopedia of Genes and Genomes
LBDD: ligand-based drug design
LD: lethal dose
LDA: Linear Discriminant Analysis
MCC: Matthews correlation coefficient
MLP: Multilayer Perceptron
MSE: mean squared error
NuBBE: Nuclei of Bioassays, Ecophysiology and Biosynthesis of Natural Products
PCC: Pearson correlation coefficient
QSAR: quantitative structure-activity relationship
QSPR: quantitative-structure property relationships
RBM: restricted Boltzmann machine
RF: random forest
ROC: Receiver Operating Characteristic
SBDD: structure-based drug design
SVM: support vector machine
T3DB: Toxin and Toxin Target Database
TC: Tanimoto coefficient
TCM: Traditional Chinese Medicine
TOPKAT: TOxicity Prediction by Komputer Assisted Technology
TPR: true positive rate
UNPD: Universal Natural Products Database
VS: virtual screening
